# Cascading Walks Model for Human Mobility Patterns

**DOI:** 10.1371/journal.pone.0124800

**Published:** 2015-04-10

**Authors:** Xiao-Pu Han, Xiang-Wen Wang, Xiao-Yong Yan, Bing-Hong Wang

**Affiliations:** 1 Institute of Information Economy, Hangzhou Normal University, Hangzhou 311121, China; 2 Alibaba Research Center for Complexity Sciences, Hangzhou Normal University, Hangzhou 311121, China; 3 Department of Physics, Virginia Polytechnic Institute and State University, Blacksburg, Virginia 24061-0435, USA; 4 Department of Modern Physics, University of Science and Technology of China, Hefei 230026, China; 5 School of Systems Science, Beijing Normal University, Beijing 100875, China; 6 Department of Transportation Engineering, Shijiazhuang Tiedao University, Shijiazhuang 050043, P. R. China; 7 Web Sciences Center, University of Electronic Science and Technology of China, Chengdu 610051, China; Beihang University, CHINA

## Abstract

**Background:**

Uncovering the mechanism behind the scaling laws and series of anomalies in human trajectories is of fundamental significance in understanding many spatio-temporal phenomena. Recently, several models, e.g. the explorations-returns model (Song et al., 2010) and the radiation model for intercity travels (Simini et al., 2012), have been proposed to study the origin of these anomalies and the prediction of human movements. However, an agent-based model that could reproduce most of empirical observations without priori is still lacking.

**Methodology/Principal Findings:**

In this paper, considering the empirical findings on the correlations of move-lengths and staying time in human trips, we propose a simple model which is mainly based on the cascading processes to capture the human mobility patterns. In this model, each long-range movement activates series of shorter movements that are organized by the law of localized explorations and preferential returns in prescribed region.

**Conclusions/Significance:**

Based on the numerical simulations and analytical studies, we show more than five statistical characters that are well consistent with the empirical observations, including several types of scaling anomalies and the ultraslow diffusion properties, implying the cascading processes associated with the localized exploration and preferential returns are indeed a key in the understanding of human mobility activities. Moreover, the model shows both of the diverse individual mobility and aggregated scaling displacements, bridging the micro and macro patterns in human mobility. In summary, our model successfully explains most of empirical findings and provides deeper understandings on the emergence of human mobility patterns.

## Introduction

In recent years, benefited from the development of location tracking technologies, the studies on the statistics of human daily mobility rise to be a new research hot point. Researchers have analyzed varieties of datasets which could reflect human mobility (e.g. dollar-bill tracking, mobile phone roaming, global positioning system, recording of taxi and bus passengers, et al). These empirical studies show two major quantitative features of human mobility patterns: i). the scaling anomalies in several statistics, which mainly include the aggregated jump-size (or say move-length or displacement) of each travel [1–8], waiting time [2, 6], visitation frequency of each distinct location [2, 6], and the growth of the total number of visited locations [2, 6]. ii). Ultraslow diffusion property, which is observed in the slow growth of the mean square displacement (MSD) and radius of gyration [1–3, 6]. Beyond that, several other anomalies, such as high regularity and predictability [9, 10] and the limitation of traffic system [5], have also been observed in human mobility. These features are not only against the traditional view of human mobility based on random walks but also sharply different from the pure Lévy-flight nature. Consequently, researchers have to review the discussions on many social and economic dynamics that are deeply affected by human mobility, such as the spreading of pathogens and information [11–16], the emergence of collective behaviors [17], the planning of urban sub-domains and traffic systems [18], and so on.

Moreover, with the progress on this issue, even some recent formed consensus are also challenged by the new findings. For instance, the aggregated distribution of long-range move-length shows widely existed scaling properties, however, this conclusion can not be extend to be the universal law of human mobility [19], since human individuals’ movements show high diversity [20] and the pattern of urban trips usually appear to be exponential-like [21–24].

A key issue is how to uncover the origin of these properties. Recent studies have proposed several approaches and dynamical models. The simplest type is the descriptive model, including the continuous-time random-walk (CTRW), which can generate power-law-like displacement distribution and show slow diffusion [1], and the self-similar least action walk (SLAW) [4]. Many possible origins have been proposed in explaining the scaling anomalies. The first type is the aggregated effect on population to mimic the macroscopic features of human mobility. Such as the radiation model that can reproduce much realistic inter-city mobility patterns [25], and the model based on maximum entropy theory under Maxwell-Boltzmann statistics which aggregates the scaling law from diverse individual movements [20]. In the second type, the spatial heterogeneity of population density and urban environment was discussed as a factor that contributes to the emergence of human mobility patterns [23, 26, 27]. Beyond these qualities, recently, the hierarchical nature of human mobility is empirically observed [7] and is considered as one of the origin of the scaling move-length [7, 28]. For the ultraslow diffusion, the model reported in Ref. [29] indicates that it seems to be the result of the high-frequency trips between homes and working locations. And some recent studies focus on the effect of social interactions and propose models based on the co-evolution between agents’ mobility and social networks, creating scaling properties on both social networks and mobility patterns [30]. Recently, the model proposed by Song et al [6] attracts much attention. It considers two generic mechanisms: the explorations of new locations and the preferential returns of former visited locations. In combination of some previous results obtained from empirical analysis, this model provides several statistical patterns consistent with the empirical data, indicating that the two mechanisms play fateful role in human mobility. Unfortunately, due to the dependence on the previous empirical results, this model does not give a sufficient explanation on the characteristic key of human mobility patterns: the scaling law on jump-size. Overall, a model that catches the key in human mobility and reproduces most of empirical observations (scaling laws and ultraslow diffusion) from few basic assumptions is still lacking.

In the present paper, considering the empirical findings of the correlations of displacements [31], based on the cascading-like process and combined with the localized exploration and preferential returns, we propose an agent-based model to explain the underlying mechanisms behind human daily travels. We find that, this minimum model can quantitatively well rebuild almost all the empirical findings and cover most of the results of previous modeling studies, implying the cascading-like process is indeed important in understanding human mobility patterns.

## Results

### The model

One of the noticeable features of our daily long-range travels is that, we usually prefer to take some short-range movements around the aiming locations before returning to the starting point. For example, when we travel from our hometown to another city, generally speaking, we need to find a hotel, then we will take some activities for the purpose of the travel (e.g., working, meeting, or tours) at certain locations near the city, and at last we return to hometown after all objectives have been completed. Each activity we take during the travel will create one or several additional short-range movements in the region of the city, that is to say, the long-range travel from our hometown to the aiming city activates a series of shorter movements in the aiming city. Similarly, such kind of activations can also be observed in many middle-range or short-range movements, for instance, the commuter’s trips vs. the movements in workplaces, and the trips from home to marketplace vs. the strolls during shopping. Associating with the activation by long-range movements, an explicit feature of human mobility emerges: a series of activating processes perform most transitions from the relatively longer travels to the shorter trips, and each of them can initiate a series of shorter trips near the aiming location of the longer travel. These serial initiations of movements are named as “Cascading Walks Process”.

This cascading walks process in human mobility have been observed by recent empirical studies based on the database of GPS trajectories [31], in which the evidences of cascading walks process that the positive correlation between the displacements of two consecutive movements, positively relates to the scaling property of human mobility patterns, implying the cascading walks process might play an important role in the emergence of human scaling mobility patterns. Also, previous studies on the temporal patterns of human activities indicated a similar cascading-like process would be relevant to the emergence of burst [32], as well as the long-term correlations (can be created by the cascading processes) [33, 34]. Overall, combining both the daily experiences and empirical studies above, the cascading walks process is regarded as the basic mechanism of our model which will be elucidated later.

**Fig 1:**
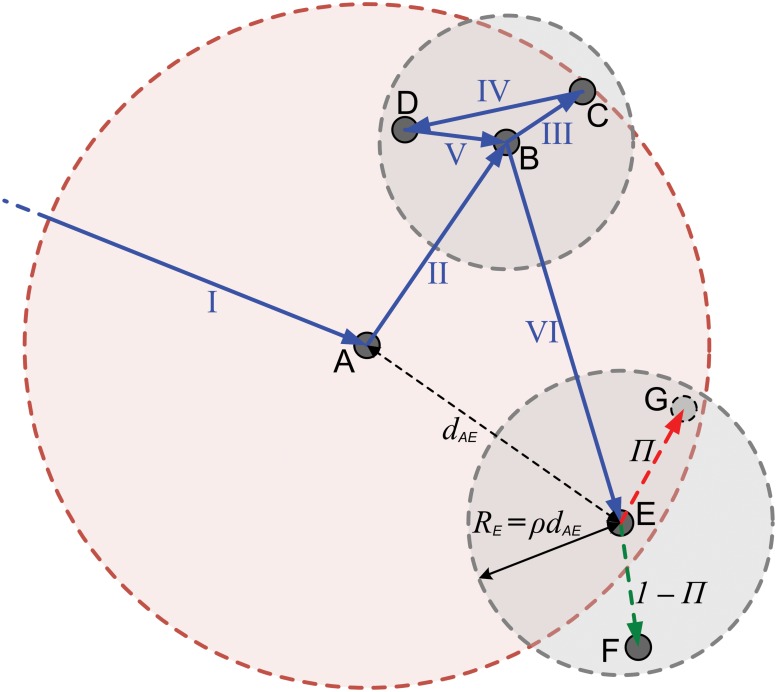
Illustration of the cascading walks process in the present model. It shows several consecutive movements of the walker, and the dashed circles represent the regions in different layers.

The initiation of a series of shorter movements after a longer travel is the basic dynamics of cascading walks process, and thereby in the present model, the longer travel and initiated shorter movements can be distinguished into two layers: the longer travel stays in the higher layer, the shorter movements are in the lower one, and the corresponding end locations also can be set into different layers. An illustration for the algorithm of the cascading walks in the present model is shown in Fig 1. Associating with Fig 1, detailed rules will be shown below.

i). The localized cascading process. It’s the basic rule of the present model. We assume that the walker moves to an *i*-th layer location A (the movement I in Fig 1). The long-range movement I is in the *i*-th layer and initiates several (*i* + 1)-th layer shorter movements in the region of A (the red dashed circle), which are marked as II (A → B) and VI (B → E), as shown in Fig 1. The two (*i* + 1)-th layer locations B and E are randomly chosen in the region of A. The movements II and VI respectively initiate several (*i* + 2)-th layer short trips in the region of B and E (the two gray dashed circles, and they are called the sub-region of A), for instance, the movements III, IV and V in the region of B. These (*i* + 2)-th layer trips could again initiate (*i* + 3)-th layer movements (it’s not shown in Fig 1). The radius *R*′*s* of the region B/E, are respectively proportional to the distances from A to B/E: *R*_*B*_ = *ρd*_*AB*_ for location B, as well as *R*_*E*_ = *ρd*_*AE*_ for E, where *ρ* is a parameter satisfying 0 < *ρ* < 1. Here it is allowed that parts of the region of B/E are outside the region of A. And during the term that the walker moves in the region of location A, say, every visited locations besides location A are set into the layer lower than location A, until the exhaustion of the staying time of A (it will be defined in the next rule), as well as other locations (B, C, ⋯).

ii). The staying time is correlated with the traveling distance. For the location B, say, the total time of all these lower-layer short movements which are directly or indirectly initiated by the movement II is *τ*_*B*_, which means *τ*_*B*_ is the time interval from the beginning of movement III to the end of movement V, or say, the staying time of B. We assume that the staying time *τ*_*B*_ is positively correlated with the distance from the up-layer location A to the location B,

$$\begin{array}{c} \tau_{B}=f_{P}\left(d_{AB}^{\gamma }\right). \end{array}$$ (1)

It means that *τ*_*B*_ is a random number which follows a Poisson distribution *f*_*P*_ with an average value of $d_{AB}^{\gamma }$, as well as $\tau_{E}=f_{t}(d_{AE}^{\gamma })$ for the location E, and similarly for other locations, for instance, $\tau_{C}=f_{P}(d_{BC}^{\gamma })$, where *γ* is another main parameter of the model, denoting how the staying time is correlated with the distance between up-layer locations. According to the empirical findings, *γ* is set to be the positive and not greater than 1, namely, the relationship of Eq (1) is mainly sub-linear. In the simulations, we set a rule that when the staying time runs out, the walker must return to the center location of the current region to prepare the departure to another region. For instance, when *t* = *t*_*B*_ + *τ*_*B*_ − 1, the walker moving in the region of B must return to B, no matter whether the staying time in the current region runs out or not. This assumption is realistic and supported by our empirical analysis results of the volunteers’ GPS records (see Section 0.1 of *Materials and Methods*), in which indeed show the above power functional correlation on the staying time in the relevant region and the moving displacement in the region, and the fitting value of *γ* is less than 1.

iii). Localized exploration and preferential return in prescribed region. During the period that the walker moves inside the region of a location (the (*i* + 1)-th layer location E, say), as shown in Fig 1, each initiation of a new (*i* + 2)-th layer movement will be of the probability

$$\begin{array}{c} \Pi =\frac{\lambda }{\lambda +\Sigma_{j}k_{j}}. \end{array}$$ (2)

The walker can visit a new (*i* + 2)-th layer location (G, say) from its present location, where G is randomly chosen in the region of E, the parameter *λ* controls the preference of explorations, and Σ_*j*_ *k*_*j*_ denotes the total number of visitations of all the (*i* + 2)-th layer locations in current (*i* + 1)-th layer region except the current location. And with probability 1 − Π, the walker can return to a former visited (*i* + 2)-th layer location (F, say), chosen with a probability that is proportional to its former visitation number *k*. In other words, in this region and this layer, the location that are visited more frequently are more possible to be visited again. Note that, the (*i* + 1)-th layer location E is also treated as one of these (*i* + 2)-th layer locations in the region, if E is not the current location, as well as A for the the (*i* + 1)-th layer locations in its region. The walks in other layers, e.g. A → B and B → E, also obey the above algorithms. Compared with the algorithm of Song’s model [6], the explorations and preferential returns are localized in the current region and current layer.

### Modeling Results

At the initial time *t* = 0 in simulations, the walker starts traveling from the initial location with coordinate (0, 0) at the top layer. The radius of the region of the initial location, which also is the maximum radius for all the regions, is fixed to be *R*_0_ = 10^4^. In the following discussions, if not specially mentioned, the value of *λ* is set to be the default value 0.5. All the statistical results that will be discussed below are obtained from the simulations with 5 × 10^4^ time steps after a relaxation with the same time steps. What’s more, instead of using the absolute value of displacement, we use the relative displacement $\Delta x_{t}=\frac{|{\vec{X}}_{t+1}-{\vec{X}}_{t}|}{R_{0}}$, where ${\vec{X}}_{t}$ and ${\vec{X}}_{t+1}$ are respectively the coordinates of the agent at time step *t* and *t* + 1.

**Fig 2:**
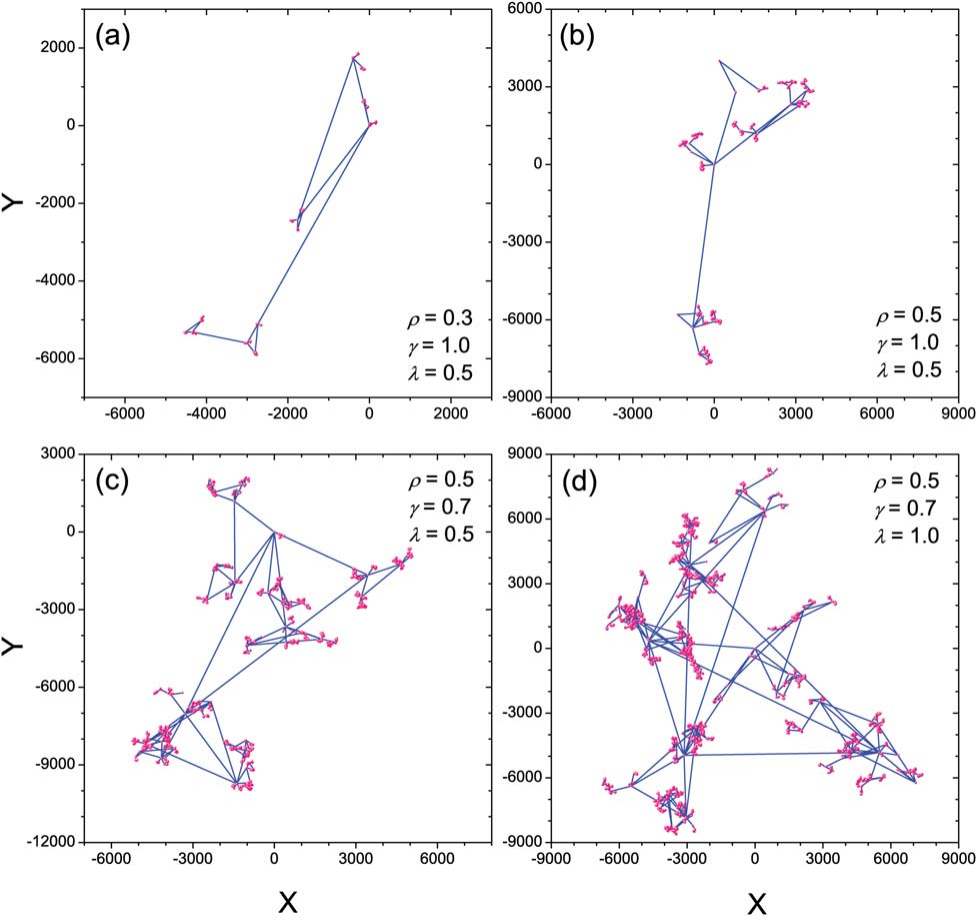
Typical trajectories of the model. Illustrations of the trajectories of 5 × 10^4^ consecutive travels generated by the model with four typical parameter settings of *ρ*, *γ* and *λ*.

The patterns of trajectories generated by the present model are very similar to our intuitive experience in daily life. Fig 2 shows the typical trajectory patterns under different parameter settings. The trajectories in different scales have significant self-similar property, and large *ρ*, small *γ* along with large *λ* can generate more complicated trajectory patterns. Most importantly, we find that the present model can create most of the properties observed in empirical studies, including both the scaling anomalies and ultraslow diffusion. In the following subsections, we will discuss them one by one.

i) Displacement distributions.

**Fig 3:**
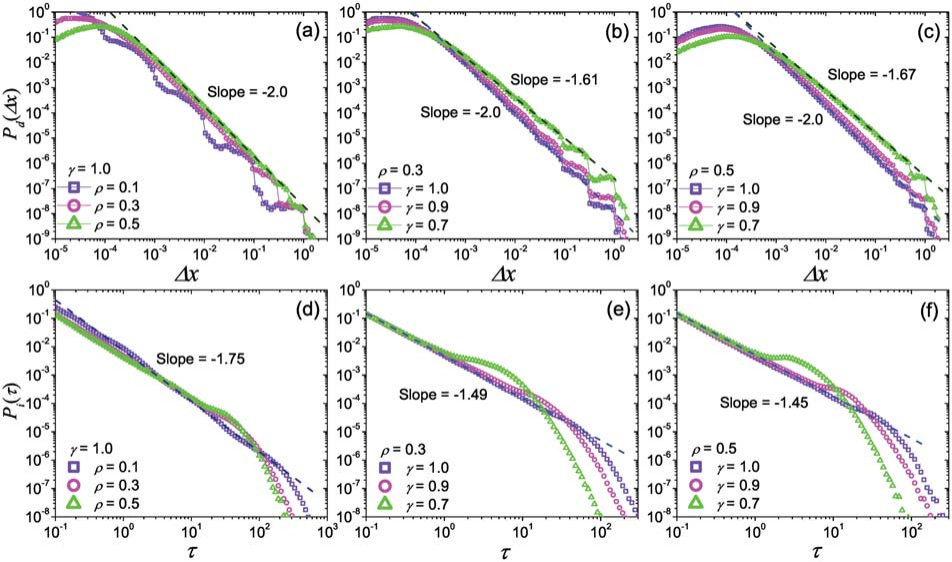
The displacement distributions and the inter-visit time distributions. Panels (a), (b) and (c) are respectively the displacement distributions generated by the present model with different parameters *ρ* and *γ*, as well as panels (d), (e) and (f) for the inter-visit time distributions. The result is averaged by 10^3^ independent runs. The dark blue dashed lines and dark green dashed lines respectively corresponds to the fitting lines of the data signed by light blue squares and light green rectangles.

The scaling statistics of move-lengths is one of the most noticeable differences between real-world human mobility patterns and the random walk nature, and has been observed in many animals movements [35–37]. Empirical studies have provided lots of evidences to support a widely accepted viewpoint that the displacement distributions of human travels at the population level are power-law-like [1, 2], no matter the arguments at the individual level. Now, understanding the origin of the scaling displacement distribution becomes an important task for modeling researches. In present model, the numerical simulations indicate that the displacement distribution with a power-law tail can emerge in all parameter space (Fig 3(a), 3(b) and 3(c)). The exponent *α* of the power-law tail is mainly affected by *γ*, and is insensitive to other parameters. The value of *α* is 2.0 when *γ* = 1.0, and will reduce along the reduction of *γ* (Fig 3(b) and 3(c)), covering the range of many previous empirical studies and our findings on GPS records (see Section 0.1 of *Materials and Methods*). When the value of *ρ* is small, even though the fluctuations on the curves of displacement distribution are obvious, it does not damage the scaling property in the tail (Fig 3(a), 3(b) and 3(c)). Moreover, we estimated the average value of *ρ* and *γ* from empirical analysis on Geo-life GPS dataset, and then run the model on this empirical-based parameter settings (*ρ* = 0.66, *γ* = 0.54). The curve of simulation results is almost parallel to the empirical one (see Section 0.1 of *Materials and Methods*). More detailed discussion can be found in Section 0.1 of *Materials and Methods*.

Simulations also show that the parameter *λ* doesn’t affect the displacement distribution at the aggregated level at all. Moreover, due to the locations is randomly chosen in region of upper layer location, the displacement distribution for the movements in single layer is homogeneous. In the real-world human mobility, the use of vehicle usually follow the travel length and thus it shows obvious hierarchy, for instance, taxi or bus for urban trips, and airplane for long-range travels. Recent studies indicated that human travels using single type of vehicle (e.g. taxi, et al) and short-distance commuting usually do not show scaling property [22, 38]. Our modeling results for single layer mobility successfully match the empirical findings. The analytical result (see Section 0.2 of *Materials and Methods*) indicates that, the exponent *α* only depends on *γ* with the expression of *α* = 1 + *γ*, and the scaling displacement distribution essentially emerges from the localized cascading process, which determines the hierarchical organization of locations. It plays an important role in the sub-linear relationship between the staying time *τ* in a certain region and the corresponding traveled displacement Δ*x*.

**Fig 4:**
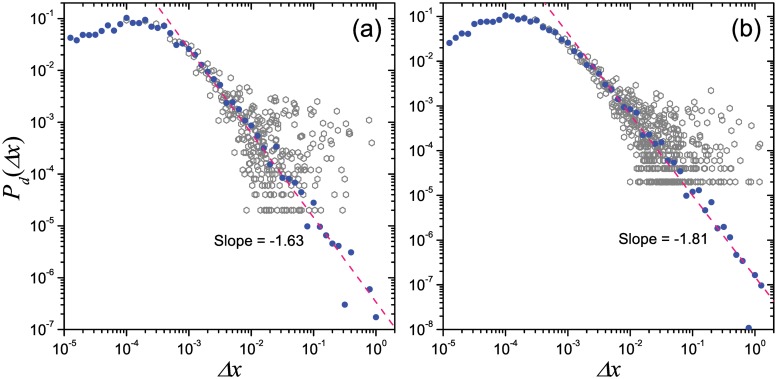
Two examples of the displacement distributions of single walker in single run. Panels (a) and (b) respectively correspond to the simulation shown in Fig 2(c) and 2(d), and blue dots and grey hexagons respectively show the log-binning distribution and its original form, and the pink dashed lines shows the fitting power law curves.

Note that, the results in Fig 3 are obtained by an average of a large number of simulations. In our simulations each run is completely independent with other runs, therefore we can consider each run as a simulation for an agent, accordingly *P*_*d*_(Δ*x*) in Fig 3(a), 3(b) and 3(c) actually shows the aggregated patterns of displacement distributions for multiple agents, which corresponds to most of empirical studies. What does the real-world travel patterns at the individual level look like is still in controversy. Recent studies reported some evidences that, human mobility shows rich diversity at the individual level, and the displacement distribution of some users would show obvious deviation from the power law [20, 29]. Our empirical analysis on Geo-life GPS dataset also provide similar properties (see Section 0.1 of *Materials and Methods*). The diversity in individual-level mobility is also observed in the present model. We plot the displacement distributions generated by single runs. Fig 4 shows two typical examples of the displacement distribution of single run, in which the original data points in tail are scattered in a large region. The log-binning curves still looks close to a power law, however, due to many of the repeated trips, the probabilities of few long-range displacements usually are 10^3^ or more times larger than the expected values on fitting power law. And also, the displacements of these repeated long-range movements for different runs (corresponds to different individuals) are usually very different. In this case, it is not surprising that a small sample deviating from power law can be abstracted from this system with intrinsical scaling property. Possibly, it is the origin of diversity on human mobility patterns at the individual level.

ii) Inter-visit time distributions.

The inter-visit time of a location is the time interval between two consecutive visits of the same location. Several earlier researches, for example, the power-law-like inter-event time distributions of library borrowing [39], implies that the inter-visiting time distribution of many locations could have non-Poisson properties. Recently, several direct observations based on cell phone and online society datasets pointed out that the inter-visiting time distribution indeed has obvious fat-tail property and usually is effected by users’ life cycles [2, 8].

Power-law-like inter-visit time distributions surprisingly emerge in our model with slopes between -1 and -2 (Fig 3(d), 3(e) and 3(f)) under different parameter settings. The curves of the inter-visit time distribution *P*_*i*_(*τ*) vary as *γ* decrease, from power-law functions to bimodal forms with power-law heads. The fitting exponent *ζ* of the head of *P*_*i*_(*τ*) is close to 1.5 and somewhat negatively correlate to the value of *ρ* (Fig 3(d), 3(e) and 3(f)), but not sensitive to *γ*. For large *ρ*, a tiny peak appears in the tail of *P*_*i*_(*τ*) and moves to the front when *γ* is small. Similar to the displacement distributions, *λ* almost doesn’t affect the inter-visit time distribution.

iii) Visitation frequency and the number of visited locations

Empirical studies of human mobility patterns have indicated that, a few locations, which usually are working places and supermarkets, are visited much more frequently than other places. This heterogeneity is described by the power-law-like visitation frequency distributions in Zipf’s ranking plot with a Zipf’s exponent *ξ* ≈ 1.2 [2, 6]. According to the relationship *ξ*′ = 1 + *ξ* ^−1^ between the Zipf’s exponent *ξ* and *ξ*′, where *ξ*′ is the exponent of the correspondingly power-law probability density function (PDF), we can get a exponent *ξ*′ of 1.8 in the PDF of visitation frequency distribution.

**Fig 5:**
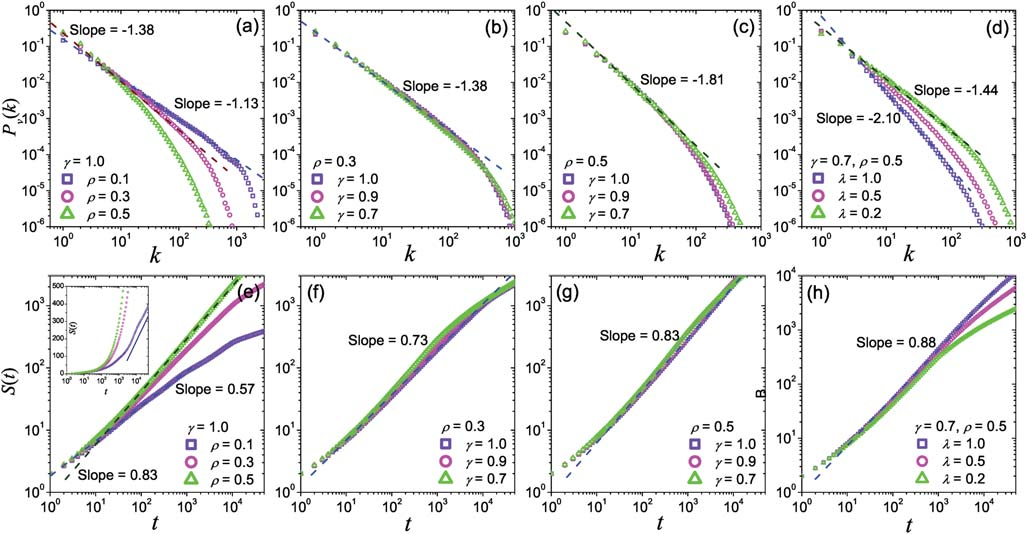
Visiting frequency distributions and the growths of the number of visited locations. Panels (a), (b), (c) and (d) are respectively the visiting frequency distributions generated by the present model with different parameters *ρ* and *γ*, as well as panels (e), (f), (g) and (h) with the growth of the number of visited locations. The result is averaged by 10^3^ independent runs. The dark blue dashed lines and dark green dashed lines respectively corresponds to the fitting lines of the data signed by light blue squares and light green rectangles, and the blue line in the inset of (e) shows the logarithmical section.

This scaling visitation frequency is reproduced by the present model. The curves in Fig 5(a)–5(d) illustrate the power-law-like PDF *P*_*v*_(*k*) of visitation frequency for locations under different parameter settings. *P*_*v*_(*k*) is very sensitive to the value of *ρ*: The fitting value of the PDF exponent *ξ*′ reduces from 1.8 to 1.1 along the growth of *ρ* from 0.1 to 0.5. The analysis in Section 0.3 of *Materials and Methods* indicates that in a extreme case without the cascading effect, *ξ*′ is 1.0, implying that larger *ξ*′ possibly originates from the cascading effect. Moreover, the preference on explorations *λ* also strongly affect *P*_*v*_(*k*): the more explorations, the larger value of *ξ*′ (Fig 5(d)).

The growth of the number of visited locations *S*(*t*) is generally expected to be in a scaling form *S*(*t*) ∼ *t* ^*μ*^. *μ* = 1 stands for the pure Lev́y flights, and its value depends on the exponent of the staying time distribution, where staying time is the time that a walker spend at one location in CRTW [9]. Empirical studies have indicated that, in real-world *S*(*t*) obeys a scaling law with an exponent of *μ* ≈ 0.6, which is smaller than the prediction in CRTW [2, 9], indicating an ultraslow growth of the number of visited locations and a growing returning probability versus the visited locations. The growth of *S*(*t*) in the present model also obeys a power-law function with an exponent *μ* < 1 (Fig 5(e)–5(h)). Similar to the situation of visitation frequency distribution, the fitting exponent *μ* of *S*(*t*) is mainly affected by the value of *ρ*, where smaller *ρ* corresponds to slower growth of *S*(*t*) (small *μ*), indicating that the walkers are more likely to return to the former visited locations for smaller *ρ*. The reason is that, smaller *ρ* limits the movements and explorations of new locations in sub-layer regions, and the walker has to frequently return to former visited locations in upper layer. The effect of *γ* and *λ* is weak. An extreme situation without the cascading effect shows a logarithmic-growing *S*(*t*) (see Section 0.3 of *Materials and Methods*), which results that *S*(*t*) with large *ρ* can generate logarithmic sections (Fig 5(e)). Thus we infer that the cascading effect deeply changes *S*(*t*) from the logarithmic form into the power-law form.

iv) Radius of gyration and MSD.

**Fig 6:**
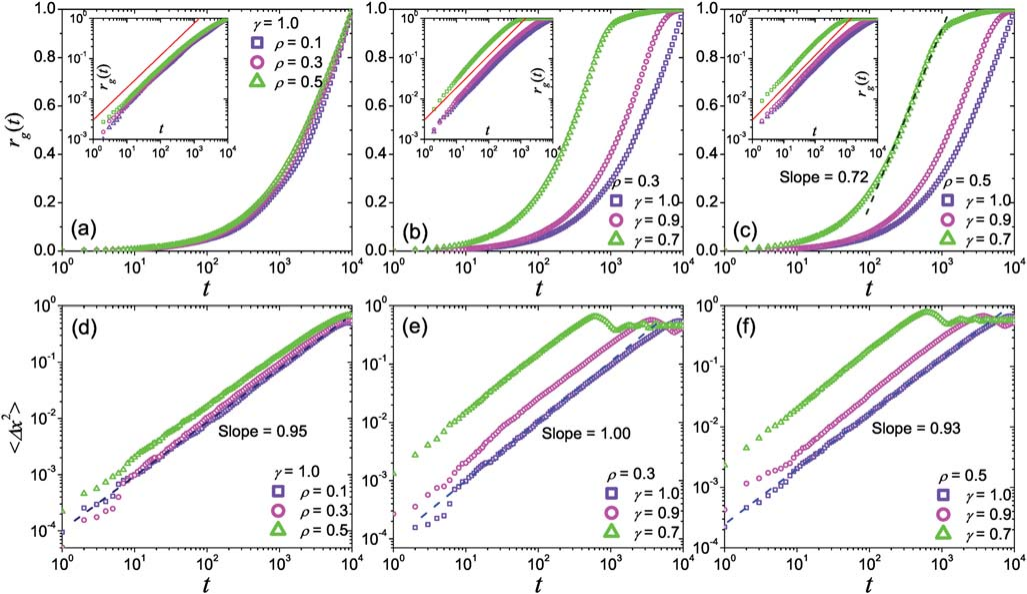
Gyration radius and MSD of the model. Panels (a), (b) and (c) are respectively the growth of radius of gyration generated by the present model with different parameters *ρ* and *γ*, as well as panels (d), (e) and (f) for the MSD. The result is averaged by 10^3^ independent runs. The insets in panels (a), (b) and (c) show the corresponding *r*_*g*_(*t*) curves in log-log plots. The dark blue dashed lines and dark green dashed lines respectively corresponds to the fitting lines of the data signed by light blue squares and light green rectangles, and the red line in each inset indicates the power function with exponent 0.80.

The properties of human spreading are mainly shown in the growth of radius of gyration *r*_*g*_ and MSD versus time. For the discrete time model, the radius of gyration of a walker in the time period (0, *t*) is defined as:

$$\begin{array}{c} r_{g}\left(t\right)=\sqrt{\frac{1}{t}\sum_{i=1}^{t}{\left({\vec{x}}_{i}-{\vec{x}}_{m}\right)}^{2}}, \end{array}$$ (3)

where ${\vec{x}}_{i}$ represents the position of each visited location, and ${\vec{x}}_{m}$ is the center of the trajectory during the period [2]. Empirical studies noted that the growth of the radius of gyration of human *r*_*g*_(*t*) is close to the logarithmic form rather than the power function predicted by the CRTW model and Lev́y flight, indicating an ultraslow spreading in human long-range travels [2, 6]. We also calculate the *r*_*g*_(*t*) of GPS carriers using Geo-life GPS datasets and find that *r*_*g*_ obviously deviates from power function for the *t* larger than 0.5 day (see Section 0.1 of *Materials and Methods*). This abnormal property can also be reproduced by the present model. Generally, the modeling *r*_*g*_(*t*) shows a gradual deviation from the initial power functional form with exponent about 0.8 (close to our empirical finding discussed in Section 0.1 of *Materials and Methods*). When *γ* < 1, *r*_*g*_(*t*) shows a partly logarithmic-like growth in the region *t* > 100, as shown in Fig 6(c), which generally is in agreement with some empirical findings on the mobility of mobile users [2, 6].

The MSD ⟨Δ*x* ^2^⟩ at time *t* is defined as:

$$\begin{array}{c} \left\langle \Delta x^{2}\left(t\right)\right\rangle =\left\langle {\left({\vec{x}}_{t}-{\vec{x}}_{0}\right)}^{2}\right\rangle \end{array}$$ (4)

where ${\vec{x}}_{0}$ provides the coordinates of initial position and ⟨⋅⟩ means the average over total running time. Previous studies indicated that, ⟨Δ*x* ^2^(*t*)⟩ ∼ *t* for Brownian random walks and ⟨Δ*x* ^2^(*t*)⟩ ∼ *t* ^1/(*α*−1)^ for pure Lévy flight, and the MSD of real-world human mobility is generally slower than the prediction of Lévy flight [3, 6].

**Fig 7:**
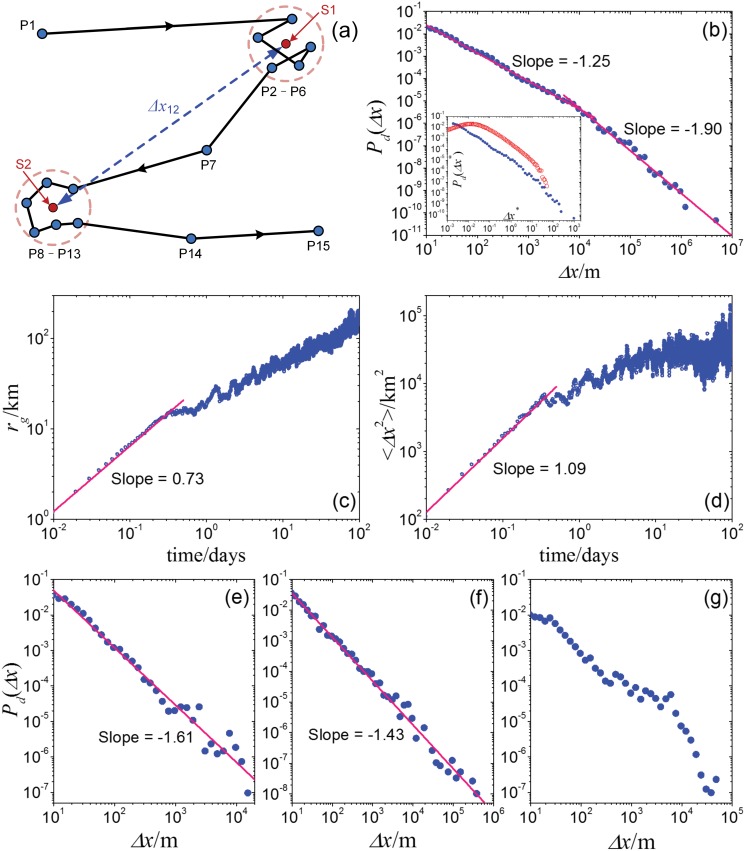
Empirical results of the analysis on GPS records. (a) Illustration of the distinguishing on effective staying positions. (b) The log-binning aggregated displacement distribution *P*_*d*_(Δ*x*), and the inset shows the displacement distributions *P*_*d*_(Δx*) of the unified displacement Δx* = Δ*x*/⟨Δ*x*⟩ for the empirical results (the blue dots) and the modeling results under parameter settings *ρ* = 0.66, *γ* = 0.54, and *λ* = 0.5 (the red hexagons). Panels (c) and (d) respectively show the gyration radius and MSD of the 32 users in aggregated level, and the unit respectively are kilometer (km) and square kilometer (km^2^). Panels (e), (f) and (g) show the log-binning displacement distributions of three of the 32 users, and the unit of displacement is meter (m).

The curves of MSD generated with different parameter settings are shown in Fig 6(d)–6(f). The fitting exponents are much close to 1.0, which is generally close to the empirical findings based on GPS carriers [3], and also can in agreement with our empirical findings on Geo-life GPS datasets (see Section 0.1 of *Materials and Methods* and Fig 7(d)). For the cases *γ* < 1, due to the fitting exponent *α* of the displacement distribution *P*_*d*_(Δ*x*) is less than 2.0, the growth of MSD in our model is slower than the prediction by the pure Lévy flight and is more similar to the empirical findings. Here, *λ* also does not show obvious effect on both *r*_*g*_ and MSD.

These results indicate that the ultraslow diffusions mainly emerge under the condition of the sub-linear relationship (*γ* < 1). In this situation, the time that the walker spends moving within the regions of lower layer locations is relatively short, and therefore the walker will present more frequent returns to the upper layer locations and consequently results in the ultraslow diffusions.

## Discussions

The basic assumption of the model is the cascading process in both the spatial and temporal correlations, which is supported by several recent empirical observations [7, 31]. For another assumption, the localized preferential returns and explorations, compared with Song et al’s model [6], its expression is linear, and its effect is localized. Apart from these two assumptions, this model does not introduce any other spatio-temporal heterogeneities, implying that the scaling properties in long-range human mobility can emerge even without the effect of environmental heterogeneity (e.g. population density, urban environments, et al) [23, 26].

This minimum model reproduces most statistics observed in the empirical analysis, including the scaling anomalies in move-length, inter-visit time and visitation frequency, the slow growth of the number of visited locations, radius of gyration and MSD. Since the scaling displacements is the most noticeable feature in real-world human mobility, the present model successfully creates it without introducing any prior scaling properties. The analytical result of this model indicates that the cascade-like process is the key of the emergence of scaling move-lengths. And also, our model indicates the important role of the sub-linear relationship between the staying time and the displacement in dynamics.

Moreover, the present model provides an unified explanation for the differences on empirical findings in different levels and bridges several controversial issues. First, it shows both the aggregated scaling law and diversity of individual mobility, in agreement with the recent empirical findings across the macroscopic and microcosmic domains [20], indicating a possible mode for the emergence of the aggregated scaling properties of large number of individuals with diverse mobility patterns. Secondly, the result that the mobility in single layer do not show the scaling jump-length, is similar to the real-world situations with single type of vehicle (e.g. bus, taxi, et al) and the short-distance commuting [22, 38], successfully explaining both the scaling anomalies on move-length in global level and the homogeneous properties in urban mobility and single-vehicle trips [20–23, 27]. In addition, the scaling visitation frequency in the model implies the existence of dominant trips, in agreement with peoples’ daily experiences and empirical findings [2, 6, 20].

In summary, the present model shows that, only based on the two basic mechanisms, the cascading walks process and the localized preferential returns and explorations, are enough to explain most of the statistics for human mobility in different levels. This conclusion would be very helpful in the understanding of the underlying mechanisms driving human mobility patterns and valuable for the prediction of human daily trips [40] and further applications.

## Materials and Methods

### 0.1 Empirical analysis and findings

To test the assumption that the staying time *τ* in a region power-functionally depends on the displacement that the walker moves into that region (see Eq (1)), we empirically analyze the real-world human mobility patterns.

We analyze the same data set that were discussed in Ref. [31]. The dataset provides the GPS records from the Microsoft Research Asia in Geo-life Project [41–43] and available online (http://research.microsoft.com/en-us/downloads/b16d359d-d164-469e-9fd4-daa38f2b2e13/). It contains the GPS records of handheld equipments and smart phones over three years (April, 2007—Sep., 2010) of 165 volunteers. In the dataset, the trajectory records of each user are of several files. Each of the trajectory files consists of a continuous sequence of tracking points, and each tracking point provides information on the latitude, longitude, and altitude of the position of the GPS holder, along with the corresponding recording time. The total recording time for different individuals is different, and ranges from several weeks to several years.

Due to GPS records can not directly show the positions that users really have stayed in, firstly we distinguish the effective staying positions from the records. Same to the method used in Ref. [31], we set the resolutions of distinguishing the staying positions to be 10 meters for the displacement which is the critical spatial resolution of a handhold GPS equipment, and 120 seconds for the time which is close to the interval of traffic signals. Consider a trajectory labelled by {*P*_1_, *P*_2_, ⋯, *P*_*N*_}, where a continuous sub-sequence {*P*_*j*_, ⋯, *P*_*k*_} (1 ≤ *j* ≤ *k* ≤ *N*) satisfies the following two conditions: the distances between two consecutive track points are less than 10 meters, and the total time length of the sub-sequence {*T*_*j*_, ⋯, *T*_*k*_} is larger than 120 seconds. The average position of the sub-sequence is recorded as an effective staying position. As illustrated in Fig 7(a), the average position S1 of track points from P2 to P6 are considered as an effective staying point, as all the geographical distances from P2 to P6 are no more than 10*m* and *T*_6_ − *T*_2_ < 120*s*. Similarly, the track points from P8 to P13 is distinguished to be another effective staying position S2. The straight-line distance between S1 to S2 is set as the user’s displacement for the movement from S1 to S2.

The analysis for users’ mobility patterns requires long-time continuous movement records. However, most of the files in this dataset only contain the records of a few minutes or hours, and we usually can not obtain enough effective stay positions to achieve good statistical patterns of user’s mobility. To get long-time continuous records, we therefore abandon all the files where the recording time is less than 6 hours, and we are left with 927 files from 100 users. Using the above distinguishing algorithms, we distinguish all the effective staying positions and get 17296 effective movements in total. Fig 7(b) shows the log-binning displacement distribution of these movements, in which the tail can be well fitted by a power law with slope −1.90, indicating the scaling property at the aggregated level. The average ratio ⟨ρ_*_⟩ between each two consecutive displacements (the shorter to the longer) is 0.44, and the real-world average value of *ρ* is estimated as 0.66 (ρ_*_ = 2*ρ*/3).

Nonetheless, in the analysis at the individual level, the number of effective staying positions from many of the 100 users is still too small to extract its patterns. We thus remain with the data of 32 users with a number of effective staying positions that is more than 200. Here we one by one analyze the files from the same user, and the results of the user are aggregated from all of his/her files. The aggregated radius of gyration and MSD of the 32 users are shown in Fig 7(c) and 7(d). The growth of radius of gyration obeys power function (the fitting slope is 0.73) within the time scale less than half-day and then trends to slow, which is slightly different from the logarithmic-like type in some previous reports [2]. MSD also shows similar growth pattern, and the fitting exponent of its front power functional region is 1.08. We calculate the displacement distributions for each of the 32 users and find that most of them can be well fitted by a power law in log bins, and the fitting power-law slopes generally are between −1 and −2, as the two examples shown in Fig 7(e) and 7(f). However, some of them show obvious deviation from strict power-law distribution, as the typical example shown in Fig 7(g), denoting the diversity on human mobility patterns at the individual level.

**Fig 8:**
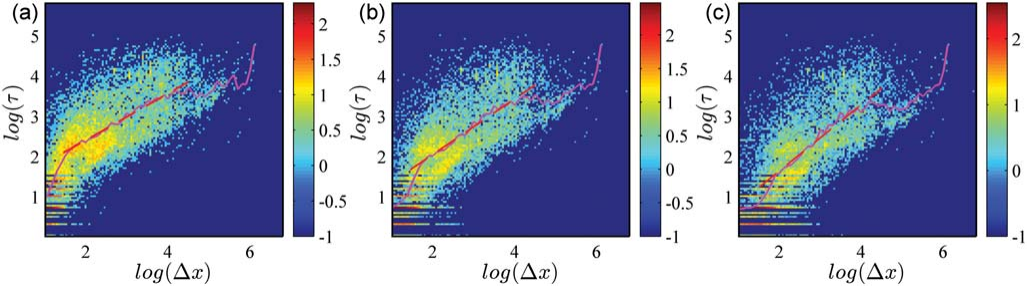
The heat map of the staying time *τ* in the region with radius *ρd* vs. the displacement Δ*x* that the user travels into the region. Here *ρ* = 0.500 (a), *ρ* = 0.250 (b) and *ρ* = 0.125 (c). The color is proportional to the logarithm of the heat (count) on each data point. The magenta curves provide the log-binning result of the relationship *τ* vs. Δ*x*, and the red dashed lines are the fitting power functions of the log-binning curves, where fitting slopes *γ* = 0.58 ± 0.01 for (a), *γ* = 0.69 ± 0.02 for (b) and *γ* = 0.82 ± 0.03 for (c).

Since the assumption of Eq (1) is one of the cores of our model, the dependence between the staying time *τ* and the corresponding displacement Δ*x* is empirically analyzed to test this assumption, where *τ* is the time interval between the moment that the user moves into a small region with radius *ρ*Δ*x* and the moment it moves out, and Δ*x* is the displacement that the user takes when moving into that region. We plot all the data points (*τ*, Δ*x*) of the 32 users’ movements and calculate the distribution of *τ* versus Δ*x*, as shown in Fig 8. For different *ρ*, the correlations are all obviously positive. And its log-binning curves show that its averaged effect generally is indeed power-functional-like and sub-linear, and the fitting exponent *γ* slightly grows from 0.58 to 0.82 along the reduction of *ρ* from 1/2 to 1/8. That is why we introduce the assumption of Eq (1) and set *γ* ≤ 1 in our model. Considering the estimated average value of *ρ* is 0.66, the corresponding fitting *γ* is about 0.54. Under this parameter settings, the displacement distribution of our model generally is parallel to the empirical curve (see the inset of Fig 7(b)), even though the size of our model limits the stretching on the tail, indicating our model can well fit the empirical findings on displacement distribution.

### 0.2 Analytical results of the displacement distributions

In the present model, according to the basic rules of the model: *R* = *ρd*_*c*_, $\tau =f_{p}(d_{c}^{\gamma })$, and assuming ${\bar{\Delta x}}_{1}=1$ for the movements in first layer, the averaged displacement in the *i*-th layer movements and the corresponding staying time can be derived immediately:

$$\begin{array}{c} {\bar{\Delta x}}_{i}=\rho_{*}^{i-1}, \end{array}$$ (5)

and

$$\begin{array}{c} {\bar{\tau }}_{i}={\bar{\Delta x}}_{i}^{\gamma }=\rho_{*}^{(i-1)\gamma }, \end{array}$$ (6)

where ρ_*_ is the ratio of the averaged radius of the (*i* + 1)-th layer and *i*-th layer regions: $\rho_{*}={\bar{R}}_{i+1}/{\bar{R}}_{i}$. Obviously, ρ_*_ = 2*ρ*/3, since the center of the (*i* + 1)-th layer is randomly chosen in the *i*-th layer region.

From Eq (2), averagely, each *i*th-layer movements can activate $\bar{\tau_{i}}/\bar{\tau_{i+1}}$ of (*i* + 1)-th layer trips. On average, we can have

$$\begin{array}{c} \Omega_{i+1}=\Omega_{i}\bar{\tau_{i}}/\bar{\tau_{i+1}}=\rho_{*}^{-\gamma }\Omega_{i}, \end{array}$$ (7)

where Ω_*i*_ denotes the summation of the probability of all the *i*-th layer movements, as well as Ω_*i*+1_ for the (*i* + 1)-th layer. The expression of Ω_*i*_ is:

$$\begin{array}{c} \Omega_{i}=\int_{0}^{\Delta x_{max}}p_{i}(\Delta x)d(\Delta x), \end{array}$$ (8)

where Δ*x*_*max*_ = 2*ρ* ^*i*−1^ denotes the maximum possible length of the movements in the *i*-th layer, and *p*_*i*_(Δ*x*) presents the probability that an *i*-th layer movement is of a displacement Δ*x*, and Ω_*i*_ satisfies the normalized condition Σ_*i*_Ω_*i*_ = 1. From Eqs (7) and (8), we can immediately have:

$$\begin{array}{c} \int_{0}^{2\rho^{i}}p_{i+1}\left(\Delta x\right)d\left(\Delta x\right)=\rho_{*}^{-\gamma }\int_{0}^{2\rho^{i-1}}p_{i}\left(\Delta x\right)d\left(\Delta x\right). \end{array}$$ (9)

Considering ${\bar{\Delta x}}_{i+1}=\rho_{*}{\bar{\Delta x}}_{i}$, and assuming that *p*_*i*_(Δ*x*) and *p*_*i*+1_(Δ*x*) have similar forms, the *p*_*i*_(Δ*x*) and *p*_*i*+1_(Δ*x*) will have following relationship:

$$\begin{array}{c} p_{i+1}\left(\Delta x\right)=Cp_{i}\left(\frac{\Delta x}{\rho_{*}}\right) \end{array}$$ (10)

where the constant *C* can be obtained approximately by Eqs (9) and (10):

$$\begin{array}{c} C\approx \rho_{*}^{-(1+\gamma )} \end{array}$$ (11)

The probability of a displacement with a relative length Δ*x* is the summation of *p*(Δ*x*) over different layers:

$$\begin{array}{c} P_{d}\left(\Delta x\right)=\sum_{i\leq j}p_{i}\left(\Delta x\right) \end{array}$$ (12)

where *j* is the maximum layer number that satisfies Δ*x* ≤ 2*ρ* ^*j*−1^, since the maximum possible length of the *j*-th layer movements is 2*ρ* ^*j*−1^.

Also due to the center of the *j*-th layer is randomly chosen in the (*j* − 1)-th layer region, the distribution *p*_*j*_(Δ*x*) is in homogeneous type, and thus we can assume that the peak of *p*_*j*_(Δ*x*) is much closer to the averaged value ${\bar{\Delta x}}_{j}$. And considering Eq (10), we have:

$$\begin{array}{c} p_{i}\left({\bar{\Delta x}}_{j}\right){|}_{i<j}<\rho_{*}^{\left(1+\gamma \right)}p_{j}\left({\bar{\Delta x}}_{j}\right) \end{array}$$ (13)

We thus can have:

$$\begin{array}{c} p_{j}\left({\bar{\Delta x}}_{j}\right)<P_{d}\left({\bar{\Delta x}}_{j}\right)<\frac{1-\rho_{*}^{j(1+\gamma )}}{1-\rho_{*}^{\left(1+\gamma \right)}}p_{j}\left({\bar{\Delta x}}_{j}\right) \end{array}$$ (14)

$P_{d}({\bar{\Delta x}}_{j})$ therefore is approximately proportional to $p_{j}({\bar{\Delta x}}_{j})$. Combining with Eq (10), and ${\bar{\Delta x}}_{j}=\rho_{*}^{j-1}$, we can get:

$$\begin{array}{c} P_{d}\left({\bar{\Delta x}}_{j}\right)\approx \rho_{*}^{-(1+\gamma )}P_{d}\left(\frac{{\bar{\Delta x}}_{j}}{\rho_{*}}\right) \end{array}$$ (15)

We therefore obtain $P_{d}({\bar{\Delta x}}_{j})\propto {\bar{\Delta x}}_{j}^{-\alpha }$, where the power law exponent *α* = *γ* + 1.

$P_{d}(\bar{\Delta x})$ for the $\bar{\Delta x}$ of different layers construct the basic power-law-like form of the displacement distribution *P*_*d*_(Δ*x*), therefore *P*_*d*_(Δ*x*) generally obeys a power law form with the exponent *α* = 1 + *γ*, even though it has slight fluctuation in the condition with small *ρ*. The analytical result is in agreement with the numerical simulations shown in Fig 3. Here the exponent *α* only depends on *γ*, nevertheless, it is in the condition where 0 < ρ_*_ < 1, indicating that the cascading process in the model is of the basic requirement in the origin for the power-law-like displacement distribution.

### 0.3 Analysis of the visitation frequency and the number of visited locations in a situation without the cascading effect

An extreme situation is the model without the cascading effect (ρ* = 0). In this case, all the movements and locations are in the same layer, and Eq (2) would be the only rule that drives the model. The evolution of the visitation frequency distribution $P_{v}^{\prime }(k^{\prime })$ can be derived directly:

$$\begin{array}{c} kP_{v}^{\prime }\left(k\prime \right)=\left(k\prime -1\right)P_{v}^{\prime }\left(k\prime -1\right), \end{array}$$ (16)

Note that, here *k*′ represents the total number of visitation of a location from the initial time *t* = 0. We obtain:

$$\begin{array}{c} P_{v}^{\prime }\left(k\prime \right)\propto k^{\prime -1}. \end{array}$$ (17)

Considering the initial process from *t* = 0 to *t* = *t*_0_, for *t* > *t*_0_, we have: $P_{v}^{\prime }(k_{0}+k)\propto {\left(k_{0}+k\right)}^{-1}$ and $P_{v}^{\prime }(k_{0})\propto k_{0}^{-1}$, where *k*_0_ and *k* respectively denote the total number of visitation of a location before and after *t*_0_. So we can have $P_{v}^{\prime }(k)\propto k^{-1}$, which is the visitation frequency distribution in this extreme situation.

For the growth of the number of visited locations *S*′(*t*), in this extreme situation, according to the definition of *S*′(*t*) and $P_{v}^{\prime }(k)$, we have

$$\begin{array}{c} S^{\prime }\left(t\right)=\sum_{k}P_{v}^{\prime }\left(k\right), \end{array}$$ (18)

and

$$\begin{array}{c} t=\sum_{k}\left[kP_{v}^{\prime }\left(k\right)\right]. \end{array}$$ (19)

Substituting $P_{v}^{\prime }(k)\propto k^{-1}$ into the two equations above, we can easily obtain that *S*′(*t*) ∝ ln*t*.

In brief, removing the cascading effect, the visitation frequency distribution of the model will obey a power law form with the exponent of −1, and the growth of the number of visited locations will show a logarithmic relationship over time.
